# The Effects of Periconceptional Risk Factor Exposure and Micronutrient Supplementation on Birth Defects in Shaanxi Province in Western China

**DOI:** 10.1371/journal.pone.0053429

**Published:** 2012-12-31

**Authors:** Wenfang Yang, Lingxia Zeng, Yue Cheng, Zhijun Chen, Xiang Wang, Xu Li, Hong Yan

**Affiliations:** 1 The First Affiliated Hospital of Medical College in Xi’an Jiaotong University, Xi’an, Shaanxi Province, P.R. China; 2 Department of Public Health, Xi’an Jiaotong University College of Medicine, Xi’an, Shaanxi Province, P.R. China; 3 The Central of Disease Control and Prevention in Xi’an, Xi’an, Shaanxi Province, P.R. China; Tabriz University of Medical Sciences, Islamic Republic of Iran

## Abstract

**Objectives:**

1) To understand the current prevalence and main types of birth defects, 2) assess the periconceptional exposure of factors associated with birth defects in Shaanxi Province, and 3) provide scientific evidence for local governments to formulate services for the primary prevention of birth defects.

**Methods:**

We sampled 16,541 households from 128 townships in 16 counties/districts in Shaanxi province using a multi-stage random sampling method. Among them, 10,544 women who had live born or stillborn infants with gestational age ≥28 weeks between 2008 and 2009 were interviewed using a structured questionnaire designed to collect information about periconceptional risk factor exposure, health care service utilization, and micronutrient supplements. Logistic regression was performed to assess the risk factors associated with birth defects and adjustments were made for imbalanced social-demographic characteristics between case and control groups.

**Results:**

The prevalence of congenital birth defect in Shaanxi province was 14.3/1000 births. The environment risk factors associated with birth defects include unhealthy lifestyle (Alcohol, odds ratio (OR): 3.60, 95% confidence interval (CI) 1.64−7.91; Smoking, OR: 1.32, 95% CI: 0.99−1.75; Drink strong tea, OR: 1.81, 95% CI: 1.27−2.59), exposure to heavy pollution (OR: 1.53, 95% CI: 1.01−2.30), maternal diseases (OR: 1.77, 95% CI: 1.35−2.33), drug use (OR: 2.11, 95% CI: 1.51−2.95), maternal chemical pesticide exposure (OR: 2.30, 95% CI: 1.16−4.57), and adverse pregnancy history (OR: 10.10, 95% CI: 7.55−13.53). Periconceptional folic acid or multiple micronutrients including folic acid supplementation, was associated with a reduced rate of birth defects (OR: 0.54, 95% CI: 0.29−0.998).

**Conclusions:**

Health care service utilization, unhealthy lifestyle factors, and environment risk factors seem to be associated with birth defects in Shaanxi province. Governmental agencies should focus on effective primary preventative methods, such as the delivery of periconceptional health education for minimizing potential risk factor exposures, periconceptional folic acid or micronutrient supplementation, environment monitoring, and assessment of factories with high levels of pollution.

## Introduction

Birth defects are a global problem, but their impact on infant and childhood death and disability is particularly severe in low- and middle-income countries. Serious birth defects can be lethal. For those who survive early childhood with birth defects, they can experience lifelong mental, physical, auditory, and visual disabilities that exact harsh human and economic tolls on them, their families, and their communities. Up to 70% of birth defects could either be prevented or, with proper care, cured or ameliorated [1], [2]. The prevalence rates of serious genetic and partly genetic birth defects are generally considered to be similar throughout the world. However, several factors contribute to variation in populations between and within countries and across regions, including poverty and health care [2], [3].

As the largest developing country in the world, a hospital-based surveillance system for monitoring birth defects was established in China in 1986 [4]. Many hospitals and institutions have generated a large number of birth defect files and publications. There are large regional differences in birth defect prevalence. At present, the rate of birth defects in Shaanxi province is higher than the Chinese average [5], even though the provincial government has made great efforts to reduce birth defects. Despite surveillance system data, additional findings from population-based cross-sectional surveys, case-control studies, and large scale clinical trials are important alternative approaches to provide supplementary evidence for policy makers to formulate health care service and public health strategies. In 2009, the provincial government began distributing free periconceptional folic acid supplements (400 µg per day) through the Family Planning System and Maternal and Child Health Care System. At the same time, the central government refocused from secondary and tertiary prevention to primary prevention, which meant moving from prenatal care to preconceptional or periconceptional care. In order to provide evidence for the local government to formulate services for the primary prevention of birth defects, a population-based study was conducted in Shaanxi Province. The primary objectives of this study were to understand the current prevalence and main types of birth defects and to assess periconceptional exposure to environmental risk factors associated with birth defects. This study also served as baseline survey for further community-based intervention studies on birth defect prevention.

## Methods

### Data Sources

A population-based cross-sectional survey on birth defect prevalence rates and types was conducted in Shaanxi Province in Western China in 2009–2010 using a stratified, multistage cluster random sampling technique. Information on live births or stillbirths with complete gestational age ≥28 weeks born in the previous 2 years was gathered from parents or caregivers during household interviews.

A sample of 13,347 household was estimated to be sufficient to detect a 1.4% birth defect rate and 20% acceptable variability in live born or stillborn infants, given assumption of α = 0.05, β = 0.20, and design effect = 2.0. The sample size was expanded to 16,016 households to account for a 20% non-response rate. Prior to fieldwork, the research team conducted village, township, and county listing operations to prepare multi-stage probability proportion to size (**PPS**) sampling. Households were selected with a two-stage sampling procedure. The 16 counties/districts in Shaanxi Province were selected during the first stage by PPS sampling. There were 125 births in each township averagely during 2008–2009 according to the data from Provincial Maternal and Child Healthcare center, so eight townships in each selected county/district were chosen during the second stage for ensure that 1000 eligible household would be covered in the survey. All of the households that met the inclusion criteria were interviewed in 128 selected townships.

### Ethical Approval

The research protocol and informed consent procedure were approved by the Human Research Ethics Committee of the College of Medicine, Xi’an Jiaotong University (No. 2009020). The project was also approved by the Population and Family Planning Committee in Shaanxi Province, China. Prior to the interview, informed written consent to participate in the study was obtained from main respondents of the household and was witnessed by the village doctor.

### Data Collection

The household questionnaires were used to collect information on the women and their most recent pregnancies in surveyed households where the women were the main respondent. The questionnaire consisted of two sections. The first was a cross-sectional survey designed to estimate the prevalence rates of birth defects in Shaanxi Province and covered the following topics: (1) respondent socio-demographic characteristics (education, occupation, age, nationality, etc.), (2) husband’s background, (3) family economic level, (4) antenatal and delivery care of the most recent pregnancy, and (5) birth outcomes of the most recent pregnancy. The second section was a case-control study that focused on the women’s reproductive history, periconceptional exposure to risk factors associated with birth defects in the most recent pregnancy, and the use of folic acid and other micronutrient supplementations. Periconceptional was defined as 3 months before pregnancy through the first 3 months of pregnancy. All the households completed the first section of the questionnaire, and the second section was completed when the mother was available to interview. One village was randomly selected in each county/district for re-interviewing within one week after the first visiting to assess the self-reporting accuracy.

### Training and Fieldwork

Training courses for field staff were carried out in two locations. The first was conducted at Xi’an Jiaotong University College of Medicine, and the second course was given in Chang’an County in Shaanxi Province. Both courses consisted of an introduction to interviewing skills and fieldwork procedures, how to complete the questionnaire, interview simulations in households outside the sample area. Data collection was carried out by interviewing teams, each of which was responsible for four counties and consisted of one team leader, eight field interviewers, one Maternal and Children Health (MCH) staff member, one officer from the county health bureau, one local driver, and one interpreter if needed. Data collection took place between August 2009 and April 2010.

### Data Processing

The data were self-checked by the field interviewers immediately after each interview and cross-checked by the team leaders for completeness and consistency of all items in the questionnaires before setting out for next village. The completed questionnaires were taken to the Center of Epidemiology and Biostatistics in Xi’an Jiaotong University College of Medicine and double entered through double data verification. The kappa statistics were used to evaluate consistency of categorical variables.

### Case Classification

All live- and stillborn infants with birth defects were selected as case subjects (International Statistical Classification of Disease and Related Health Problems 10th Revision ICD-10, congenital malformations, deformations, and chromosomal abnormalities Codes Q00–Q99). Information on infants with birth defects were cross checked with birth outcome records at the local Family Planning Station to ensure that the information was collected correctly. The control subjects were live born infants without birth defects and their mothers were available to interview.

### Exposure Assessment

Information on maternal illness and drug use, use of periconceptional multiple micronutrient supplements, and risk factor exposures [6] was obtained through household interviews of case and control parents. Maternal illness was defined as any kind of disease during pregnancy, including infectious disease, diabetes [7], [8], anemia, sexually transmitted disease, hepatitis, thyroid disease, acute fatty liver, gestational intrahepatic cholestasis, and pregnancy-induced hypertension. Drug therapy including antibiotics (e.g.: ampicillin, norfloxacin), anti-cancer drugs (e.g.: Vinorebine), anti-tuberculosis (TB) drugs (e.g.: retozide, pyrazinamide ), anti-thyroid drugs, hormones (including sex hormones) (e.g.: progesterone), anti-diabetic drugs (e.g.: insulins), anti-epileptic drugs (e.g.: phenobarbitoil), salicylic acid drugs (e.g.: acetaminophen), anti-hypertensive drugs (e.g.: cilazapril), anti-trichomonas fungal drugs (e.g.: metronidazole), serum products, and toxoid products were considered as irrational drug use [6], [9], [10], [11], [12]. Risk factors were divided into following categories: (1) unhealthy lifestyle including alcohol use (drinking Chinese wine or beer with frequency more than once per week) [6], [13], tobacco use/secondhand smoke exposure (smoking cigarette or staying in smoke environment with Continuous or cumulative three months or more) [6], [14], and drinking strong tea (with frequency more than once per week) during pregnancy; (2) distance from the residence to heavy pollution factories including coal mines, paper mills, cement plants, and coal-fired power plants [6], [15]; (3) chemical pesticide exposure (any exposure during pregnancy including insecticides, herbicides, rodenticides) [6]; (4) x-ray exposure; (5) maternal adverse pregnancy history (outcomes including miscarriage, stillbirth, any birth with birth defects, hydatidiform mole, preterm delivery, or any birth with low birth weight).

### Relative Risk Estimation

A logistic regression model was used to estimate odds ratios (OR) of risk factors and 95% confidence intervals (CIs) adjusting for imbalanced demographic characteristics between case and control groups as potential confounders, including maternal education, maternal occupation, paternal education, family income, and residence type. We used Stata version 9.2 (Stata/SE 9.2 StataCorp, College Station, TX, USA) for all analyses.

## Results

We conducted interviews in 16,541 households in Shaanxi Province with live born or stillborn infants during 2008–2009 (Table 1). Among them, 237 (14.3/1000 births) infants were diagnosed with birth defects. The distribution of defects is shown in Table 2. The most frequent specific diagnoses were congenital heart disease (56/237). Totally, 194 mothers with average 12 household in each village were re-interviewed to assess the self-reporting accuracy. The ranges of kappa statistics for 70 key categorical variables were 0.42 to 0.87 with all p values <0.05.

**Table 1:** Characteristics of households in the cross-sectional birth defect study and case (infants with birth defects)-control (infants without birth defects) study in Shaanxi Province, China 2008–2009.

Characteristic		Cross-sectional study	Case-control study	
		(n = 16541)	Cases(n = 221)	Controls(n = 10523)
Nationality	n(%)			
	Han	15860 (99.1)	220 (99.5)	10445 (99.3)
	Minority	151 (0.9)	1 (0.5)	78 (0.7)
Types of residence*	n(%)			
	Urban	3590 (22.6)	21 (9.5)	1850 (17.6)
	Rural	12317 (77.4)	200 (90.5)	8651 (82.4)
Number of family members	Mean (SD)	4.4 (1.4)	4.6 (1.5)	4.5 (1.4)
Maternal age (years)	Mean (SD)	26.2 (4.3)	26.4 (4.7)	26.1 (4.3)
Paternal age (years)	Mean (SD)	28.5 (4.6)	28.5 (5.0)	28.3 (4.6)
Maternal education*	n(%)			
	Primary	1592 (10.0)	44 (19.9)	1256 (12.0)
	Secondary	10271 (64.7)	146 (66.1)	6729 (64.1)
	High School+	4019 (25.3)	31 (14.0)	2511 (23.9)
Paternal education*	n(%)			
	Primary	981 (6.2)	31 (14.1)	737 (7.0)
	Secondary	10509 (66.4)	144 (65.5)	6973 (66.5)
	High School+	4340 (27.4)	45 (20.5)	2775 (26.5)
Maternal Occupation*	n(%)			
	Farmer	8873 (53.6)	161 (72.9)	6812 (64.7)
	Others	7668 (46.4)	60 (27.1)	3711 (35.3)
Paternal occupation	n(%)			
	Farmer	13071 (79.0)	157 (71.0)	7612 (72.3)
	Others	3470 (21.0)	64 (29.0)	2911 (27.7)
Family incomes (RMB/per month per person)* n(%)				
	<200	2947 (27.5)	79 (36.7)	2827 (27.8)
	200–500	3773 (35.2)	76 (35.3)	3592 (35.3)
	500–1000	1766 (16.5)	22 (10.2)	1685 (16.6)
	1000–3000	1805 (16.8)	30 (14.0)	1663 (16.3)
	>3000	438 (4.1)	8 (3.7)	407 (4.0)

Numbers/percentage may not sum to 100% due to missing values.

* p<0.05 between case and control group.

**Table 2:** Classification of birth defects in in Shaanxi Province, China, 2008–2009.

Congenital malformation classification	n	Rate (per 1000 births)
**Isolated cases**	233	14.09
Circulatory system	56	3.39
Congenital heart defect	56	3.39
Nervous system	32	1.93
Neural tube defects	11	0.66
Congenital hydrocephalus	14	0.85
Cerebral palsy	7	0.42
Eye, ear, face, and neck	37	2.24
Eye congenital malformations	21	1.27
Ear congenital malformations	16	0.97
Cleft lip and/or cleft palate	9	0.54
Respiratory system	3	0.18
Digestive system	11	0.67
Genital organs	5	0.30
Urinary system	2	0.12
Musculoskeletal system	23	1.39
Limb malformation	12	0.73
Other	53	3.20
Downs syndrome	1	0.06
**Multi-malformed cases**	**4**	**0.24**
**Total cases**	237	14.33

The overall participation rate in the Shaanxi Province Birth Defects Case-Control Study was 64.6% (10,523/16,294) for mothers of control infants and 93.2% (221/237) for mothers of infants with birth defects. Most demographic characteristics were balanced between the two groups. Households with case infants were more likely to live in rural areas and have lower family incomes and parental education levels compared with control infants (Table 1).

### Associations between Unhealthy Behaviors and Birth Defects

Compared with the control group, infants whose mothers drank alcohol, smoked or were exposed to secondhand smoke, and drank strong tea during pregnancy were at increased risk for birth defects (Alcohol, OR: 3.60, 95% CI 1.64−7.91; Smoke, OR: 1.32, 95% CI: 0.99−1.75; Strong tea, OR: 1.81, 95% CI: 1.27−2.59). These data are summarized in Table 3.

**Table 3:** Exposure to risk factors associated with birth defects in case and control groups in Shaanxi Province, China, 2008–2009.

Risk factor exposure		Cases (n = 221)	Controls (n = 10523)	OR (95% CI)*	P
Proximity to heavy pollution factories					
	Yes	28 (12.7)	915 (8.7)	1.53 (1.01–2.30)	0.044
	No	193 (87.3)	9608 (91.3)	1	
Alcohol					
	Yes	8 (3.6)	90 (0.9)	3.60 (1.64–7.91)	0.001
	No	212 (96.4)	1.352 (99.1)	1	
Smoke					
	Yes	143 (64.7)	6052 (57.5)	1.32 (0.99,1.75)	0.056
	No	78 (35.3)	4471 (42.5)	1	
Strong tea					
	Yes	39 (17.6)	1118 (10.6)	1.81 (1.27–2.59)	0.001
	No	182 (82.4)	9405 (89.4)	1	
History of adverse pregnancy					
	Yes	83 (37.6)	572 (5.4)	10.10 (7.55–13.53)	<0.001
	No	138 (62.4)	9951 (94.6)	1	
Chemical pesticides					
	Yes	11 (5.0)	178 (1.7)	2.30 (1.16–4.57)	0.018
	No	209 (95.0)	10328 (98.3)	1	
X-Ray					
	Yes	1 (0.5)	30 (0.3)	1.65 (0.22–12.24)	0.624
	No	219 (99.5)	10475 (99.7)	1	
Illness					
	Yes	130 (58.8)	4540 (43.3)	1.77 (1.35–2.33)	<0.001
	No	91 (41.2)	5951 (56.7)	1	
Irrational drug use					
	Yes	48 (21.8)	1163 (11.1)	2.11 (1.51–2.95)	<0.001
	No	172 (78.2)	9320 (88.9)	1	

* Adjusted by maternal education, maternal occupation, residence type, paternal education, and family income.

### Associations between Disease, Drug Use, and Birth Defects

Infants born to mothers with any kind of disease, including infectious disease, diabetes, anemia, sexually transmitted disease, hepatitis, thyroid disease, acute fatty liver, gestational intrahepatic cholestasis, and pregnancy-induced hypertension were at higher risk for birth defects (OR: 1.77, 95% CI: 1.35–2.33). The risk was greater for infants whose mothers received drug therapy, including antibiotic, anti-cancer drugs, anti-TB drugs, anti-thyroid drugs, hormones (including sex hormones), anti-diabetic drugs, anti-epileptic drugs, salicylic acid drugs, anti-hypertensive drugs, anti- trichomonas fungal drugs, serum products, toxoid products, and vaccines during pregnancy (OR: 2.11, 95% CI: 1.51–2.95) (Table 3).

### Associations between Other Risk Factor Exposures and Birth Defects

Maternal exposure to chemical pesticides (OR: 2.30, 95% CI: 1.16–4.57); households near heavy pollution factories, such as coal mines, paper mills, cement plants, and coal-fired power plants (OR: 1.53, 95% CI: 1.01–2.30); and maternal adverse pregnancy history (OR: 10.10, 95% CI: 7.55–13.53) were significantly associated with birth defects (Table 3).

### Associations between Healthcare Service Use, Micronutrient Supplementation, and Birth Defects

This study showed that there was low utilization of antenatal care check in Shaanxi Province, but the difference of proportions of antenatal care check with frequency ≥6 times and the initial antenatal care check at first trimester between the case (28.5%) and control (33.3%) groups was not statistically significant. The percentages of infants whose mothers consulted the doctor about pre- and postnatal care were 24.2% in the case group and 26.5% in the control group. Just 28.5% of infants in the case group and 33.0% of infants in the control group were brought in for at least 6 antenatal care checks, with the initial visit in the first trimester (Table 4).

**Table 4:** Health service utilization and micronutrients supplementation in case and control groups in Shaanxi Province, China, 2008–2009.

Item		Cases (n = 221)	Controls (n = 10523)	OR (95% CI)*	P
Pregnancy consultation					
	Yes	51 (24.2)	2733 (26.5)	0.98 (0.71–1.36)	0.901
	No	160 (75.8)	7568 (73.5)	1	
Antenatal care check					
	Yes	63 (28.5)	3474 (33.0)	0.97 (0.71–1.32)	0.837
	No	158 (71.5)	7049 (67.0)		
Folic acid supplementation					
	Yes	12 (5.5)	1184 (13.8)	0.54 (0.29–0.998)	0.049
	No	207 (94.5)	9269 (88.7)	1	
Iron supplementation					
	Yes	12 (5.5)	627 (6.0)	0.81 (0.30–2.21)	0.684
	No	207 (94.5)	9826 (94.0)	1	
Multiple micronutrient supplementation					
	Yes	10 (4.6)	424 (4.1)	1.34 (0.70–2.56)	0.374
	No	209 (95.4)	10029 (95.9)	1	

* Adjusted by maternal education, maternal occupation, residence type, paternal education, and family income.

The proportion of women who reported periconceptional micronutrient supplements was very low; just 11.2% of women took folic acid or multiple micronutrients supplements that included folic acid from 3 months before pregnancy through the first 3 months after pregnancy (consumption amount ≥60 tablets). We found that there was a significant impact of periconceptional folic acid intake on birth defects compared with the control group (OR: 0.54, 95% CI: 0.29–0.998) (Table 4).

## Discussion

The prevalence of congenital birth defects in Shaanxi province was 14.3/1000 births, which was slightly higher than the data (13.5/1000 births) compiled by the Birth Defects Surveillance System Network [5]. The most common were congenital heart disease. The environment risk factors associated with birth defects in Shaanxi Province include unhealthy lifestyle (alcohol, smoking, and strong tea), living near heavy pollution factories, maternal diseases, irrational drug use, maternal chemical pesticide exposure, and maternal adverse pregnancy history. We also found low utilization of health services and very low periconceptional micronutrient supplementation, even though folic acid is known to reduce the incidence of birth defects.

Estimations of birth defect prevalence in China have been derived from hospital monitoring systems since the 1980s. Hospital-based birth defect surveillance has been a useful approach for monitoring distribution and changes in birth defect incidence rates. Surveillance data provides information for epidemiological studies and support for health policy decisions, health service planning, and preventative programs [16]. However, data from hospital-based birth defect surveillance tend to underestimate the prevalence of birth defects due to the low proportion of rural household delivering in hospitals and the limited surveillance time window from 28 gestational weeks to 7 days after delivery; thus, they should not be relied upon as the sole data source for epidemiological birth defect studies. Population-based sampling surveys can provide useful data to identify causes or risk factors [16], such as drugs, nutritional factors, environmental exposures, maternal illnesses, and genetic factors. Moreover, they provide data to inform the development and evaluation of prevention strategies. The sample sites of this large-scale sampling survey covered different levels of economic development and areas with different terrain features. The survey population was limited to women of childbearing age in the past 2 years, and the data from this survey contributed to our understanding of birth defect rates and types and specific risk factors in Shaanxi, a western Chinese province with higher prevalence of birth defects.

### Shifting Birth Defect Disease Spectrums

Compared with 20 years ago, the rank of disease of birth defects in Shaanxi Province has changed, and there has been no significant reduction in the overall prevalence of birth defects. The four most common birth defects in 1996–2000 in Shaanxi Province were congenital malformations of central nervous system and neural tube defects, cleft lip and/or cleft palate, limb deformities, and congenital heart disease [17]. In this study, we found that congenital heart diseases were the most common, which is in line with global reports on birth defects.

### Specific Risk Factors for Birth Defects

In this study, we found that maternal periconceptional exposure to general risk factors, including alcohol use, drugs, chemical pesticides, and maternal illnesses, were significantly associated with birth defects, which is consistent with findings from other studies [7], [9], [10], [11], [12], [13], [18], [19]. Specific risk factors associated with birth defects in Shaanxi Province were secondhand tobacco exposure, strong tea drinking, and living near heavy pollution factories. More than half of pregnant women were exposed to secondhand tobacco smoke, about 10% of pregnant women were living near heavy pollution factories that have serious impacts on the surrounding environment [15], and more than 10% of women reported drinking strong tea during pregnancy. These findings highlight the importance of focusing on health care to policy makers.

### Periconceptional Micronutrient Supplement Use

Just 5% of pregnant women reported taking iron or multiple micronutrient supplements in Shaanxi province, despite high rates of anemia (>35%) and inadequate or deficient folate levels (>60%) among women of childbearing age, mainly due to low dietary intake of nutrients [20], [21]. A cluster randomized controlled trial in Shaanxi province previously reported that antenatal iron-folic acid supplement increased the duration of gestation, reduced early preterm delivery (<34 weeks), and was associated with a significant reduction in early neonatal mortality compared to folic acid only, especially in poor households [22], [23]. This is important given that approximately 30% of early neonatal deaths are attributable to birth defects [3]. Use of periconceptional folic acid or a multiple micronutrient supplementation that includes folic acid is still very low, even though the provincial government has provided folic acid free of charge since 2009. Despite the low use rate, folic acid supplementation has reduced the rate of birth defects. In addition to folic acid, periconceptional multiple micronutrient supplementation has been shown to further reduce birth defect rates as reported by many different studies [18], [24], [25], [26], [27], [28], [29].

This study highlighted health care service underutilization, which merits urgent attention. Just one in four women received counseling about “Good Child Rearing,” which was the main approach to provide health education regarding preventing birth defects. One in three women had ≥6 antenatal care visits and an initial antenatal care check in first trimester, which is an important critical period for embryonic development.

### Conclusions

Health care service utilization, unhealthy lifestyle, and environmental risk factors seem to be associated with birth defects in Shaanxi province. Governmental agencies should focus on implementing a series of effective primary prevention methods, such as providing periconceptional health education for controlling for potential risk factor exposures, periconceptional folic acid or micronutrient supplementation, and environmental monitoring.
